# Cardiac effects of seasonal ambient particulate matter and ozone co-exposure in rats

**DOI:** 10.1186/s12989-015-0087-3

**Published:** 2015-05-06

**Authors:** Aimen K Farraj, Leon Walsh, Najwa Haykal-Coates, Fatiha Malik, John McGee, Darrell Winsett, Rachelle Duvall, Kasey Kovalcik, Wayne E Cascio, Mark Higuchi, Mehdi S Hazari

**Affiliations:** Environmental Public Health Division, US EPA, 109 TW Alexander Drive, Research Triangle Park, Durham, NC 27711 USA; Human Exposure and Atmospheric Sciences Division, US EPA, 109 TW Alexander Drive, Research Triangle Park, Durham, NC 27711 USA

**Keywords:** Ambient particulate matter, Ozone, Season, Cardiac, Health effects, Co-exposure, Electrocardiogram, Rats, Source apportionment, Elements

## Abstract

**Background:**

The potential for seasonal differences in the physicochemical characteristics of ambient particulate matter (PM) to modify interactive effects with gaseous pollutants has not been thoroughly examined. The purpose of this study was to compare cardiac responses in conscious hypertensive rats co-exposed to concentrated ambient particulates (CAPs) and ozone (O_3_) in Durham, NC during the summer and winter, and to analyze responses based on particle mass and chemistry.

**Methods:**

Rats were exposed once for 4 hrs by whole-body inhalation to fine CAPs alone (target concentration: 150 μg/m^3^), O_3_ (0.2 ppm) alone, CAPs plus O_3_, or filtered air during summer 2011 and winter 2012. Telemetered electrocardiographic (ECG) data from implanted biosensors were analyzed for heart rate (HR), ECG parameters, heart rate variability (HRV), and spontaneous arrhythmia. The sensitivity to triggering of arrhythmia was measured in a separate cohort one day after exposure using intravenously administered aconitine. PM elemental composition and organic and elemental carbon fractions were analyzed by high-resolution inductively coupled plasma–mass spectrometry and thermo-optical pyrolytic vaporization, respectively. Particulate sources were inferred from elemental analysis using a chemical mass balance model.

****Results**:**

Seasonal differences in CAPs composition were most evident in particle mass concentrations (summer, 171 μg/m^3^; winter, 85 μg/m^3^), size (summer, 324 nm; winter, 125 nm), organic:elemental carbon ratios (summer, 16.6; winter, 9.7), and sulfate levels (summer, 49.1 μg/m^3^; winter, 16.8 μg/m^3^). Enrichment of metals in winter PM resulted in equivalent summer and winter metal exposure concentrations. Source apportionment analysis showed enrichment for anthropogenic and marine salt sources during winter exposures compared to summer exposures, although only 4% of the total PM mass was attributed to marine salt sources. Single pollutant cardiovascular effects with CAPs and O_3_ were present during both summer and winter exposures, with evidence for unique effects of co-exposures and associated changes in autonomic tone.

**Conclusions:**

These findings provide evidence for a pronounced effect of season on PM mass, size, composition, and contributing sources, and exposure-induced cardiovascular responses. Although there was inconsistency in biological responses, some cardiovascular responses were evident only in the co-exposure group during both seasons despite variability in PM physicochemical composition. These findings suggest that a single ambient PM metric alone is not sufficient to predict potential for interactive health effects with other air pollutants.

**Electronic supplementary material:**

The online version of this article (doi:10.1186/s12989-015-0087-3) contains supplementary material, which is available to authorized users.

## Background

Attempts to quantify the cardiovascular health burden resulting from exposure to particulate matter air pollution and identify biologically plausible mechanisms of action have been encumbered by the variability in PM composition. The physical and chemical properties of ambient PM in a given air shed are dependent on a number of factors including local geography, proximity to emission sources, time of day, and meteorology. Recent studies point to a pronounced seasonal pattern in fine PM (PM with an aerodynamic diameter less than 2.5 microns; PM_2.5_) composition across the U.S. with higher levels of sulfate, aluminum and magnesium in the summer and higher levels of nitrate, zinc and nickel in the winter [1]. Time-series data from 202 U.S. counties [2] point to a commensurate seasonal impact on health effects including stronger PM_2.5_ associations with same day cardiovascular and respiratory hospital admissions during the winter than in the summer. By contrast, Goldberg et al. [3] found carbon particle-related mortality was higher during the summer than in winter. In mouse studies, exposure to winter ambient PM caused greater pulmonary neutrophil influx [4] and greater systemic pro-inflammatory and procoagulant responses [5] than exposure to summer PM. In contrast, Happo et al. [6] found that PM collected in spring produced greater inflammatory responses in mouse lung than PM samples collected in the fall. Divergent responses have also been noted using *in vitro* models [7,8]. Components and/or properties of PM that vary across season and that drive season-dependent health effects of exposure need to be defined.

While the characteristics of PM are critical, PM is only one component of a complex air pollution mixture that also includes gases and volatile compounds. Assessment of the health effects of exposure at a given ambient air shed, therefore, must account for non-PM components and the potential for additive, synergistic or antagonistic responses resulting from gas-particle interactions. A growing body of evidence is pointing to interactive effects of exposure with a variety of air pollutants, including nitrogen dioxide (NO_2_), ambient PM, and O_3_ [9]. PM and O_3_ co-exposure has been linked to more pronounced cardiovascular responses including increased diastolic blood pressure [10] and dispersion of ventricular repolarization [11] in humans and decreased HRV [12], and epicardial adipose tissue inflammation in rats [13]. The unique physicochemical characteristics of PM within each season may determine interaction between components within an air pollution mixture and serve as an important contributing factor in health outcomes.

Like other regions of the U.S., central North Carolina is subject to seasonal shifts in PM_2.5_ composition with summer PM dominated by sulfate, and winter by nitrates [14]. Little is known about the influence of season on both ambient PM chemistry and cardiovascular responses within this region, particularly in the context of co-pollutant exposures. We have previously shown that exposure to various air pollutants causes exaggerated cardiovascular responses in rats [15-18]. The purpose of this study was to compare the impacts of a single summer exposure to CAPs with or without O_3_ on cardiovascular responses in rats to similar exposures during the winter and relate the responses to differences in seasonal PM composition. ECG intervals and amplitudes, HR, spontaneous arrhythmia and HRV, an indicator of autonomic tone, were measured. In addition, sensitivity to myocardial calcium loading, an index of latent vulnerability to cardiac arrhythmia, and pulmonary and systemic indicators of inflammation were assessed one day after exposure. PM exposure characteristics and meteorological conditions were documented. Finally, elemental analysis data were used to quantify PM sources using a chemical mass balance model.

## Results

### Weather patterns

**Figure 1:**
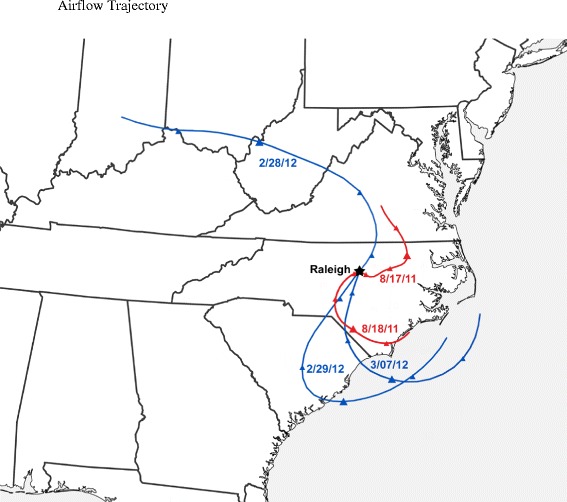
A map of superimposed backward trajectories using the Hybrid Single Particle Lagrangian Integrated Trajectory Model (HYSPLIT) at the Real-Time Environmental Applications and Display sYstem (READY) website developed by the Air Resources Laboratory of the National Oceanic and Atmospheric Administration. Archived meteorological data was utilized to model the direction and location of the test air mass for the previous 24 hr before 10 am local time (2 pm UTC (Universal Time Coordinated)) of each exposure day. Each trajectory represents a different air mass for each winter exposure day (2/28/12, and 2/29/12, and 3/07/12 and blue in color) and each summer exposure day (8/17/11 and 8/18/11 and red in color) and terminates at the exposure facility in A-Building of the US EPA campus in Durham, NC (indicated by a star). Triangle symbols indicate time of day (UTC) in 6 hour increments with larger symbols corresponding to midnight UTC (8 pm local time) in each path.

Summer and winter local weather patterns during exposures were unstable with unusually low ambient PM concentrations. Wind patterns shifted up to 180 degrees each day and were never consistent long enough to establish stable ambient PM levels. A map of superimposed backward trajectories (Figure 1) was created using the Hybrid Single Particle Lagrangian Integrated Trajectory Model (HYSPLIT; web version; Draxler, RR et al.) at the Real-Time Environmental Applications and Display sYstem (READY; Rolph, DG) website developed by the Air Resources Laboratory of the NOAA. Archived meteorological data (online) was utilized to model the direction and location of the test air mass in 6 hr increments for the previous 24 hr before 10 am local time of each exposure day. The trajectories terminated at the exposure facility in A-Building of the CRF in RTP, NC. (Latitude: 35.88350; Longitude: −78.87460). The tracings shown in Figure 1 illustrate how a uniform pattern of exposure sources was precluded because the direction of the air mass source changed for each daily exposure. Trajectories plotted do not follow air mass chronological fate after an exposure day, but rather, show how new air masses replaced current from day to day.

### Exposure concentrations

Due to unusually low ambient PM levels and unseasonably small PM sizes for the winter test days, coupled with splitting CAPs system output between 2 chambers, target PM concentrations were not achievable during the winter exposures. The protocol was revised such that for the initial PM exposures of each group, maximum achievable concentration was provided. For the subsequent exposures, we attempted to match the first day’s PM levels by including dilution air to regulate chamber PM levels. Filter samples were collected from each chamber and the system inlet for the PM exposures only as sampling the filtered air chambers (O_3_ only and air controls) provided insufficient sample mass for either accurate weighing or detectable levels for chemical analysis. One filter, 37 mm Teflon for mass concentration in the CAPs + O_3_ chamber on 8/17/11 failed during the exposure so that gravimetric sample was lost. The loss of the 37 mm Teflon mass sample did not affect PM chemical analysis or source apportionment modeling, which were performed using data derived from 47 mm Teflon, Nylasorb, and quartz filter samples. Chamber PM mass concentration for that exposure was determined by computing an average DustTrak (DT) calibration factor (DT cal factor = Filter conc/Avg DT conc) for other runs of PM filters vs average DT indicated concentrations and applying the factor to DT data for the run with the failed filter sample. Average ambient PM levels from filter samples were ~13.7 ug/m^3^ for summer and ~8.4 ug/m^3^ for winter PM exposure days. Summer ambient source PM size was larger than winter PM size producing better concentrating effects in the CAPs system during summer exposure days (Table 1). Background O_3_ levels measured in the CAPs only and air control chambers ranged from 2.5 to 10.5 ppb in the summer and winter. Chamber CAPs and O_3_ concentrations and environmental conditions for each group are reported in Table 1.

**Table 1:** **Exposures concentrations, particle size and chamber and ambient weather conditions**

**Exposure dates**	**Summer exposures**				**Winter exposures**			
	**Air**	**CAPs**	**O**_**3**_	**CAPs + O**_**3**_	**Air**	**CAPs**	**O**_**3**_	**CAPs + O**_**3**_
	23 Aug11	17 Aug11	23 Aug11	17 Aug11	5 Mar12	28 Feb12	05 Mar12	28 Feb12
	2 Aug11	18 Aug11	24 Aug11	18 Aug11	6 Mar12	29 Feb12	06 Mar12	29 Feb12
						7 Mar12		7 Mar12
**O3 (ppb)**	5.8	4.8	197.1	203.1	2.5	10.5	199.4	198.3
**PM Mass (μg/m** ^**3**^ **)**	---	168.7	---	174.9	---	78.5	---	91.3
**Particle Size (nm)**	---	333.4	---	315.0	---	125.5	---	125.0
**Geometric Mean**	---	316.1	---	295.8	---	125.5	---	125.0
**Mode**	---	316.1	---	295.8	---	125.5	---	125.0
**Geometric SD**	---	1.96	---	1.97	---	1.95	---	2.02
**Chamber Temp (F)**	72.6	72.5	70.8	70.4	73.6	72.3	71.2	69.6
**Chamber RH (%)**	51.3	50.3	51.3	56.5	46.7	42.6	51.0	47.1
**Ambient Temp (F)**	77.5	80	77.5	80	44.8	55.5	44.8	55.5
**Ambient RH (%)**	55	69.5	55	69.5	45.8	68.2	45.8	68.2

### Chemical composition of CAPs

Group-averaged PM elemental and carbon fraction composition for summer and winter exposures are shown in Tables 2 and 3. Two sets of units are required to describe the aerosol: μg PM component per g PM (Table 2) shows seasonal differences in PM composition, and μg PM component per cubic meter chamber air (Table 3) shows chamber concentrations during exposures. When expressed as μg/g, PM composition differences between summer and winter were pronounced. No elements were enriched in summer relative to winter. Relative to summer, sodium, magnesium, and calcium were enriched at levels of 4–6 times higher in winter PM, and levels of another 15 elements were enriched by 2- 3-fold. However, expressing PM concentration in units of μg/m^3^ exposure atmosphere takes into account the PM mass concentrations, which were approximately 2 times greater in summer. Table 3 shows that the overall elemental levels in the inhalation chambers were largely the same for both seasons’ exposures, with a few exceptions. Chromium and sulfate levels were 2 to 3 times higher in summer, while alkali elements sodium, magnesium, and calcium were 2 to 3 times higher in winter.

**Table 2:** **Group-averaged PM elemental composition in μg/g**

	**Summer**		**Winter**		**Winter/Summer**
**Element (μg/g)**	**CAPs**	**CAPs + O**_**3**_	**CAPs**	**CAPs + O**_**3**_	**Enrichment factor**
**Al**	951	622	2303	2250	2.9
**As**	14.8	14.2	20.3	15.5	1.2
**Ba**	304	101	335	239	1.4
**Ca**	1159	1033	4548	4200	4.0
**Cd**	2.4	2.3	9.2	6.9	3.5
**Co**	7.6	1.9	3.6	5.3	0.9
**Cr**	39.6	44.8	56.6	38.4	1.1
**Cu**	848	360	683	540	1.0
**Fe**	5053	4695	14946	10027	2.6
**K**	3637	5018	10392	7128	2.0
**Li**	6.3	11.6	32.6	21.3	3.0
**Mg**	386	271	2304	1690	6.1
**Mn**	442	688	2273	1481	3.3
**Mo**	10.3	6.7	17.7	7.8	1.5
**Na**	2404	3899	17638	13795	5.0
**Ni**	17.8	18.0	48.2	31.3	2.2
**P**	312	78	234	494	1.9
**Pb**	133	79	220	157	1.8
**SO**_**4**_	276531	294486	240898	160234	0.7
**Sb**	39.8	27.9	88.8	63.4	2.2
**Se**	29.0	37.4	50.3	40.8	1.4
**SiO**_**2**_	3822	1705	11341	6179	3.2
**Sn**	33.7	18.2	66.3	48.3	2.2
**Sr**	14.1	7.5	38.4	29.6	3.1
**Ti**	140	80	279	242	2.4
**V**	9.8	12.6	43.0	32.5	3.4
**Zn**	367	534	1475	1128	2.9
**Organic C**	287078	341624	448280	357174	1.3
**Elemental C**	14582	25329	49299	34830	2.1

**Table 3:** **Group-averaged PM elemental levels in μg/m** ^**3**^

	**Summer**		**Winter**		**Winter/Summer**
**Element (μg/m** ^**3**^ **)**	**CAPs**	**CAPs + O**_**3**_	**CAPs**	**CAPs + O**_**3**_	**Enrichment factor**
**Al**	0.160	0.109	0.181	0.205	1.4
**As**	0.00249	0.00248	0.00159	0.00142	0.6
**Ba**	0.0513	0.0176	0.0263	0.0218	0.7
**Ca**	0.195	0.181	0.357	0.383	2.0
**Cd**	0.00041	0.00040	0.00072	0.00063	1.7
**Co**	0.00128	0.00034	0.00028	0.00048	0.5
**Cr**	0.00669	0.00784	0.00444	0.00351	0.5
**Cu**	0.143	0.0630	0.0536	0.0493	0.5
**Fe**	0.852	0.821	1.173	0.915	1.2
**K**	0.614	0.878	0.816	0.651	1.0
**Li**	0.00107	0.00204	0.00256	0.00194	1.4
**Mg**	0.0651	0.0475	0.1809	0.1543	3.0
**Mn**	0.0745	0.1203	0.1784	0.1352	1.6
**Mo**	0.00174	0.00117	0.00139	0.00071	0.7
**Na**	0.406	0.682	1.385	1.259	2.4
**Ni**	0.00301	0.00316	0.00378	0.00285	1.1
**P**	0.0526	0.0136	0.0184	0.0451	1.0
**Pb**	0.0225	0.0138	0.0173	0.0144	0.9
**SO**_**4**_	46.7	51.5	18.9	14.6	0.3
**Sb**	0.00671	0.00488	0.00697	0.00579	1.1
**Se**	0.00488	0.00654	0.00395	0.00373	0.7
**SiO**_**2**_	0.645	0.298	0.890	0.564	1.5
**Sn**	0.00568	0.00318	0.00521	0.00441	1.1
**Sr**	0.00238	0.00132	0.00301	0.00270	1.5
**Ti**	0.0237	0.0140	0.0219	0.0221	1.2
**V**	0.00165	0.00220	0.00337	0.00297	1.6
**Zn**	0.0619	0.0933	0.1158	0.1030	1.4
**Organic C**	48.43	59.75	35.19	32.61	0.6
**Elemental C**	2.46	4.43	3.87	3.18	1.0
**OC/EC ratio**	16.6		9.7		

### Source apportionment analysis

**Figure 2:**
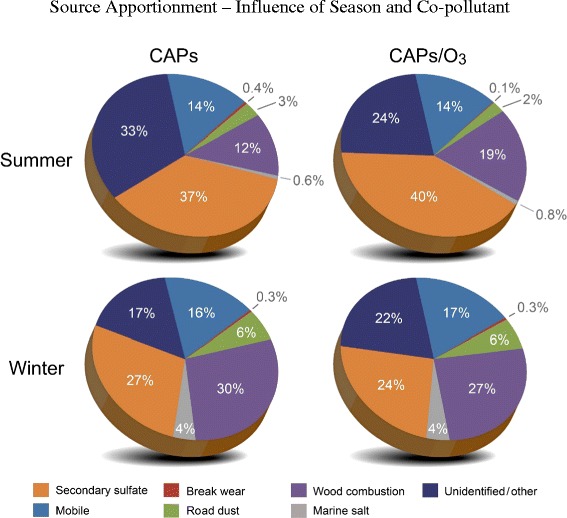
Pie Charts illustrating sources contributing to the concentrated ambient particulate (CAPs) and CAPs + ozone (O_3_) mixtures during the summer and winter exposures. Sources for each season were quantified using the EPA Chemical Mass Balance Model.

Average source contributions to the CAPS and CAPS + O_3_ mixtures are displayed in Figure 2 (Additional file 1: Table S1 and Additional file 2: Figure S1, respectively, show the same data in tabular and bar chart format); daily samples used to calculate averages are also found in Additional file 3: Table S2 and Additional file 4: Table S3, respectively. Across both seasons and pollutant mixtures, the CMB model explained between 60-100% of the particle mass (R^2^ ranged from 0.76 to 0.92). The fit statistics including percent mass explained, R^2^, chi Square, and T-statistic were within the recommended values [19]. Nitrate data were not available for inclusion in the CMB runs; therefore, some of the unresolved mass may be due to secondary nitrate (Aneja et al. [14] showed that nitrate levels in PM peak during winter months in North Carolina). The largest sources during both the summer and winter exposures included mobile sources (14-17% by mass), wood combustion (12-30%), and secondary sulfate (24-40%). Higher contributions of wood combustion were found during the winter exposures. Road dust and marine salt contributions were also higher during the winter exposures. In particular for marine salt, air flow trajectories (Figure 1) show that air masses originated from the ocean on 2/29/12 and 3/7/12. These observations are supported by the CMB results in which marine salt contributions were elevated on those two days for both the CAPS and CAPS + O_3_ mixtures (Table S2).

### Heart rate

**Figure 3:**
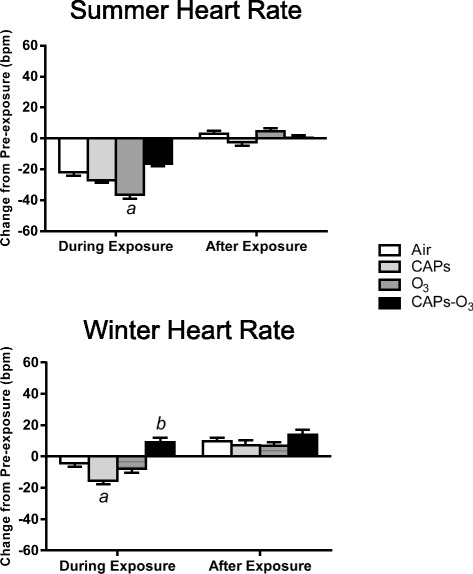
Mean change in Heart rate (HR) from pre-exposure values during summer and winter exposures. HR values for each animal at each time point during exposure or after exposure were subtracted from corresponding time-matched pre-exposure baseline data, which was recorded while the animals were either in the chamber (for “during exposure” data) or in their home cages (for “after exposure” data). Values represent mean change in HR in beats per minute ± standard error of the mean (n = 6). a - significantly less than filtered air control (p < 0.05). b - significantly greater than filtered air control (p < 0.05).

Despite being acclimated to the exposure chambers on non-exposure days, rats routinely demonstrate elevated HR during baseline pre-exposure periods on exposure days. This was evident in rats exposed to filtered air during both summer and winter months. Figure 3 shows group averages normalized to baseline pre-exposure values (i.e. Exposure minus Pre-exposure Baseline). In the summer, rats during exposure to O_3_, had a greater decrease in HR than rats exposed to filtered air (p < 0.05). There were no significant effects of CAPS or CAPS + O_3_ exposure relative to filtered air controls.

In the winter, unlike O_3_ exposure during the summer, O_3_ did not cause a significant decrease in HR during exposure. By contrast, CAPs exposure caused a greater decrease in HR than filtered air exposure (p < 0.05). CAPS + O_3_ caused the opposite effect resulting in an increase in HR relative to filtered air (p < 0.05).

HR did not change after exposure (i.e. 6-hour period after exposure) when compared to baseline in any exposure group during either season.

### Electrocardiographic parameters

**Figure 4:**
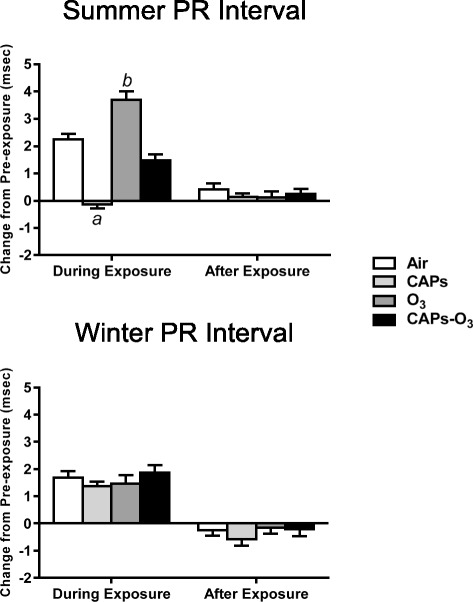
Mean change in PR interval from pre-exposure values during summer and winter exposures. PR values for each animal at each time point during exposure or after exposure were subtracted from corresponding time-matched pre-exposure baseline data, which was recorded while the animals were either in the chamber (for “during exposure” data) or in their home cages (for “after exposure” data). Values represent mean change in PR interval in msec ± standard error of the mean (n = 6). a - significantly less than filtered air control (p < 0.05). b - significantly greater than filtered air control (p < 0.05).

In the summer, O_3_ exposure significantly increased PR interval relative to filtered air controls (p < 0.05) (Figure 4). By contrast CAPS exposure significantly decreased the PR interval relative to filtered air controls (p < 0.05). CAPS + O_3_ exposure produced no effect. During winter, no exposure affected the PR interval. In addition, PR interval did not change after exposure in any exposure group during either season.

**Figure 5:**
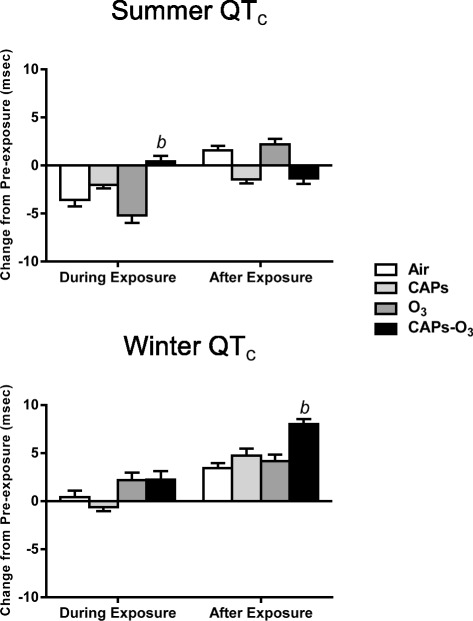
Mean change in QT_c_ interval from pre-exposure values during summer and winter exposures. QT_c_ values for each animal at each time point during exposure or after exposure were subtracted from corresponding time-matched pre-exposure baseline data, which was recorded while the animals were either in the chamber (for “during exposure” data) or in their home cages (for “after exposure” data). Values represent mean change in QT_c_ interval in msec ± standard error of the mean (n = 6). b - significantly greater than filtered air control (p < 0.05).

In the summer, there were no effects of CAPs or O_3_ exposure on QT_c_ interval relative to filtered air (Figure 5). By contrast, CAPS + O_3_ exposure caused a significant increase in QT_c_ interval relative to filtered air (p < 0.05). There was no significant effect in QT_c_ interval in the winter during exposure. However, CAPS + O_3_ exposure caused a significant increase in QT_c_ interval relative to filtered air in the winter after exposure.

### Heart rate variability

**Figure 6:**
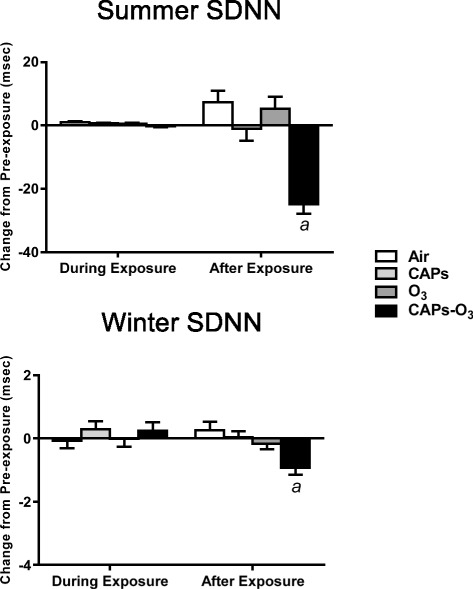
Mean change in SDNN from pre-exposure values during summer and winter exposures. SDNN values for each animal at each time point during exposure or after exposure were subtracted from corresponding time-matched pre-exposure baseline data, which was recorded while the animals were either in the chamber (for “during exposure” data) or in their home cages (for “after exposure” data). Values represent mean change in SDNN in msec ± standard error of the mean (n = 6). a - significantly less than filtered air control (p < 0.05).

During both summer and winter, there were no effects of CAPs or O_3_ exposure alone on SDNN relative to the response in filtered air controls during both seasonal exposures (Figure 6). By contrast, CAPs + O_3_ exposure during both summer and winter caused a significant decrease in SDNN during exposure relative to filtered air (p < 0.05). CAPs + O_3_ exposure during the winter also caused a significant decrease in SDNN after exposure relative to filtered air (p < 0.05), although the magnitude change was smaller compared to the effect of co-exposure during the summer.

CAPs + O_3_ exposure during the winter also caused a significant decrease in RMSSD and a significant increase in LF/HF after exposure relative to filtered air controls (p < 0.05; Additional file 5: Table S4). O_3_ exposure alone also caused an increase in LF/HF relative to filtered air (p < 0.05).

### Cardiac arrhythmia

There was no significant effect of exposure on the total number of arrhythmias relative to filtered air exposed controls with any exposure group during either season (data not shown).

### Sensitivity to aconitine One Day after exposure

**Figure 7:**
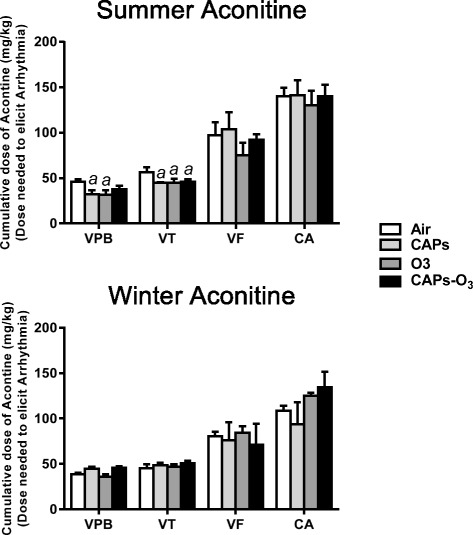
Cumulative dose of infused aconitine necessary to trigger ventricular premature beats (VPB), ventricular tachycardia (VT), ventricular fibrillation (VF), and cardiac arrest (CA) in rats one day after a single exposure. Values represent mean dose ± standard error of the mean (n = 5). a - significantly less than filtered air control (p < 0.05).

During the summer, exposure to CAPs alone or O_3_ alone significantly increased sensitivity to triggering of arrhythmia by escalating doses of aconitine as evidenced by a decrease of the total dose of aconitine necessary to elicit the first ventricular premature beat relative to filtered air exposed controls (p < 0.05, Figure 7). CAPs, O_3_, and CAPS + O_3_ each significantly decreased the total dose of aconitine necessary to elicit the first episode of ventricular tachycardia relative to filtered air exposed controls (p < 0.05). There was no significant effect of exposure on the total dose of aconitine necessary to elicit the first episode of ventricular fibrillation or cardiac arrest relative to air exposed controls.

There was no significant effect of exposure on acontine arrhythmia sensitivity with any exposure group during the winter. Interestingly, in a comparison of the aconitine doses required to assess vulnerability to arrhythmia across season, the dose required to trigger arrhythmias in air-exposed rats during the winter was significantly lower than air-exposed rats during the summer.

### Indicators of inflammation and injury in serum, plasma, and bronchoalveolar lavage fluid one day after exposure

**Figure 8:**
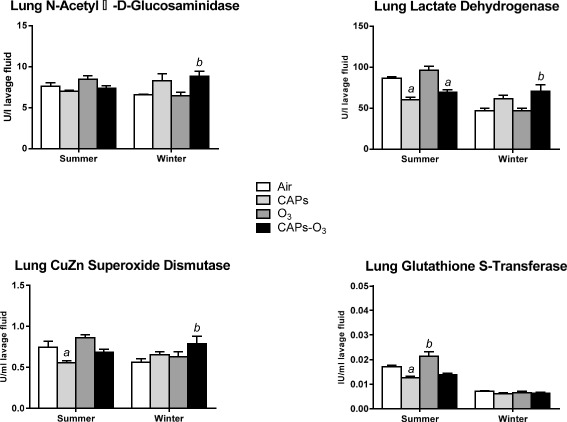
N-acetyl B-D glucosaminidase, lactate dehydrogenase, CuZn superoxide dismutase, and glutathione S-transferase in lung lining fluid one day after summer or winter exposures to concentrated ambient particulate (CAPs), ozone (O_3_), CAPs + O_3_, or filtered air. Bars represent means ± SEM for each marker shown (n = 6/group). a - significantly less than filtered air control p < 0.05). b - significantly greater than filtered air control (p < 0.05).

Exposure to CAPs + O_3_ during the winter significantly increased N-Acety-D-glucosaminidase (NAG) and lactate dehydrogenase (LDH) levels in the lung ling fluid (p < 0.05) relative to filtered air (Figure 8). There were no such changes in NAG in any of the exposure groups during the summer. In addition, exposure to CAPS or CAPs + O_3_ during the summer significantly decreased lung LDH levels (p < 0.05) relative to filtered air. CAPs + O_3_ exposure during the winter also significantly increased lung CuZn super oxide dismutase (CuZn SOD) levels (p < 0.05) relative to filtered air. There was no effect of co-exposure on CuZn SOD during the summer, whereas, CAPs exposure alone significantly decreased CuZn SOD levels (p < 0.05) relative to filtered air. Exposure to CAPs alone significantly decreased Glutathione S-Transferase (GST) levels during the summer (p < 0.05) relative to filtered air, whereas O_3_ alone significantly increased lung GST levels (p < 0.05) relative to filtered air. There were no such changes in GST in any of the exposure groups during the winter. There was no significant effect of exposure in any other indicator of inflammation in the lung, serum, or plasma or number of infiltrated inflammatory cells in the lung lining fluid (data not shown).

## Discussion

Here we present evidence that season and ozone are effect modifiers for ambient PM-induced cardiovascular responses in Spontaneous Hypertensive (SH) rats, a strain of rat known to demonstrate exaggerated cardiovascular effects in response to inhaled ambient PM, diesel exhaust particles, and acrolein [20-22]. Seasonal differences in PM were consistent with earlier findings [14], with new evidence of enrichment for anthropogenic emissions and marine salt sources during winter season exposures compared to summer exposures in central North Carolina. Single pollutant cardiovascular effects with CAPs and O_3_ were present during both summer and winter exposures, with evidence of unique effects of co-exposures with associated changes in autonomic tone during both seasons.

Biogenic emissions, agricultural activity, vehicular traffic, production of secondary organic aerosols and local weather conditions largely determine the concentration and chemical composition of North Carolina’s particulate pollution [14]. The physiochemical characteristics of PM from the Research Triangle Park, NC air shed were highly variable between summer and winter seasons. Differences included particle mass concentrations, size, organic:elemental carbon ratios, and sulfate levels and are consistent with previous measurements [14]. Although winter exposures had 50% less total PM mass than summer exposures (85 vs. 171 μg/m^3^), enrichment of metals on a μg/g basis in winter PM resulted in equivalent summer and winter metal exposure concentrations. These findings were based on data from a limited number of exposure days (two summer CAPs exposure days and three winter CAPs exposure days); future studies will need to include more PM exposure sampling days to increase robustness of analysis. Nevertheless, subsequent source apportionment analysis revealed that winter exposures were enriched primarily by wood combustion and to a lesser extent by road dust and marine salt sources compared to summer exposures. The relative impact of the enrichment of marine salt sources is unclear given that only 4% of the total PM mass was attributed to marine salt sources. PM within or near the ultrafine range (0.1 to 0.6 μm) dominates emissions from residential wood combustion while the composition of the PM is dependent on combustion conditions and furnace type, with multiple elements including potassium, sodium, magnesium, aluminum, and zinc, and transition metals like chromium and nickel present in appreciable quantities [23,24]. Exposure to PM rich in transition metals has been associated with increased cardiovascular mortality [25]. Moreover, the proportion of anthropogenic metals is a critical determinant of biological responses to PM including increased pulmonary effects on days with higher metal concentrations [26-28]. As well, summer PM, although having higher mass, may have been diluted by less bioactive compounds such as sulfates, which were present at higher concentrations during the summer. In addition, winter particle size was roughly one-third the size of summer particles (125 vs. 324 nm). Smaller, near-ultrafine particles have greater relative surface area than larger particles at any given mass concentration and have the capacity to penetrate deep into the lung and also permeate plasma membranes of cells including alveolar epithelial cells and capillary endothelium [29]. Furthermore, ultrafine PM may initiate inflammatory responses in the lung or systemic circulation or enter the blood stream and directly trigger vascular responses, either of which may trigger downstream cardiac responses [29]. In mice exposed to ambient PM collected near a highway [30] and a peat wildfire [31], size-segregated, ambient ultrafine PM caused cardiac changes, while coarse and fine PM from the same PM sample affected primarily pulmonary endpoints.

Single pollutant cardiac effects were mostly observed with summer exposures. With summer O_3_ exposure, the effects included decreased HR, increased PR interval, an increase in the HRV parameter LF/HF and increased sensitivity to myocardial calcium loading, consistent with our previous findings [16]. Summer CAPs exposure decreased PR interval and elevated sensitivity to myocardial calcium loading. The opposing PR response with summer CAPs exposure compared to summer O_3_ exposure implicates pollutant specific disparate effects on atrioventricular conduction, although each pollutant ultimately caused increased vulnerability to ventricular arrhythmia. The relevance of the increased responses to aconitine during the summer is uncertain given that exposures to CAPs and/or O_3_ during the winter had no effect on this endpoint, the precise reason of which is unclear. Nevertheless, winter time exposures caused a CAPs-induced decrease in HR, and a CAPs + O_3_-induced increase in lung lactate dehydrogenase and n-acetyl B-D glucosaminidase and increased Cu-Zn superoxide dismutase, although the relevance of these findings are also uncertain given the decrease in some of these endpoints with single pollutant exposure. The decrease in HR with CAPs alone during the winter is similar to the findings of Rohr et al. [32] who compared the cardiac effects of winter and summer CAPS exposures in Michigan in the same rat strain used in the present study. Their study had additional effects that were not found in the present study with CAPs alone including linkage of the winter HR response with increased HRV and opposing summer effects characterized by increased HR and decreased HRV. The differences between the Rohr et al. study and the present study may reflect in part the differences in study design (the Rohr et al. study consisted of thirteen 8-hour exposure days vs. a single 4-hour exposure in the present study), exposure concentrations (e.g. summer CAPs concentrations in Rohr et al. averaged ~518 μg/m^3^; in present study, ~170 μg/m^3^) and/or local contributing air pollution sources, among other factors. Moreover, the variability in the single pollutant responses in the present study may reflect an insufficient short-term exposure required to elicit a significant inflammatory response in the SH rat. This possibility is consistent with epidemiological and human exposure data that suggest that cardiovascular effects to short-term exposures are more likely triggered by autonomic responses while long-term exposures increase the likelihood of persistent inflammatory responses [29].

With some parameters during both the summer and winter seasons, CAPs + O_3_ exposure elicited unique responses that were not evident with exposure to either pollutant alone. These included changes in SDNN, RMSSD and QT_c_, during the summer, and changes in HR, SDNN, QT_c_, and lung injury and oxidative responses during the winter. The changes in QT_c_ and HR during summer and winter exposures, respectively, were each accompanied by a decrease in SDNN, which reflects overall heart rate variability, and indicates a decrease in parasympathetic tone. In addition, summer co-exposures caused post-exposure decreases in SDNN and RMSSD, another indicator of parasympathetic tone. Thus, decreased parasympathetic tone with co-exposure to CAPs and O_3_ during both seasons reflects a shift in autonomic balance towards increased sympathetic tone. Summertime CAPs plus O_3_ co-exposure in a recently conducted study in Michigan [12], characterized by greater ambient concentrations of SO4, Ni, V, Zn, and organic and elemental carbon than the present study, yielded similar HRV findings in rats. The present findings are also consistent and with other reports linking low HRV with exposure to PM [33-36] and O_3_ [37,38]. Much of the evidence on the prognostic significance of HRV points to increased cardiovascular risk with low HRV, including increased risk of arrhythmia [39] and an increased mortality rate in people with heart disease [40,41].

The autonomic responses with co-exposure may have been triggered by activation of irritant nerve fibers, including pulmonary C fibers, which trigger reflex cardiopulmonary responses via transient receptor potential (TRP) channels. We recently showed that TRPA1 mediates the increased sensitivity to aconitine-induced arrhythmia after diesel exhaust exposure [22]. In addition, Taylor-Clark and Undem [42] demonstrated that O_3_ exposure activates airway C fibers expressing TRPA1. Summer and winter CAPs exposures were rich in adsorbed elements including Fe and Ni, which have been linked to production of reactive oxygen species [7]. The potential involvement of TRP receptors in biological responses induced by transition metals should be examined in future studies given that reactive oxygen species are known activators of TRP receptors [43].

While the specific class of components that drove the observed cardiopulmonary responses is not certain, the similarity in elemental levels in the winter and summer PM due to elemental enrichment presents a plausible explanation for the physiological responses observed. More proinflammatory or irritating byproducts generated through atmospheric interaction between PM, its components, and gases might potentiate cardiovascular effects directly or via the alteration of dose distribution in the lung. For example, Fe and V salts increase the pulmonary irritancy of SO_2_ via formation of the more irritating sulfate [44]. Moreover, PM including zinc oxide and inert carbon black, when mixed with gases such as SO_2_ or O_3_, cause greater than additive pulmonary effects by potentially serving as a carrier for the reactive gases enabling greater distribution into the deep lung [44]. Further research is required to define plausible mechanisms and identify the nature of interactions between specific components like metals and gases.

Seasonal differences in PM mass concentration and size in this study precludes a direct comparison of the physiological responses to ambient PM exposure between seasons. Although air flow patterns likely had little influence, low ambient PM levels and a lower ratio of fine to ultrafine particles during the winter prevented achievement of target concentrations comparable to summer levels with our concentration system. Therefore, one approach to control for seasonal differences in mass is to lower target concentrations. Moreover, the size of the particle is naturally an outcome of the type of local air pollution sources, the level of air pollution and weather conditions. Future studies that focus on exposure to ultrafine PM alone can obviate this concern.

## Conclusions

These findings collectively point to marked seasonal effects on PM sources, mass, size, composition, and corresponding impacts on cardiovascular responses. While there was variability in some responses in this study, particularly sensitivity to aconitine-induced arrhythmia and indicators of inflammation/injury, there was evidence of electrocardiographic and autonomic responses that were unique to the CAPs and O_3_ co-exposure group. This study, however, was limited by the small number of PM sampling and exposure days, which precluded drawing conclusions about effects of entire seasons. This is especially relevant given that winter conditions often induce stable inversions that increase levels of ambient PM. Moreover, because PM composition data was obtained from only a few samples in the present study, an analysis of a potential association between cardiovascular responses and specific components of PM was not possible. In addition, the absence of nitrogen species data provides some uncertainty in the source apportionment analysis. Future studies that examine the interactive effects of CAPs and O_3_ should also mirror realistic seasonal O_3_ concentrations. Nevertheless, some evidence of unique co-pollutant effects during both seasons suggests that a multipollutant approach to health effects assessment is warranted. Furthermore, seasonal variability in sources of anthropogenic emissions and evidence for winter elemental enrichment and smaller particle size suggest that PM size and composition may be as important as mass in determining its potential for interactive health effects with other pollutants.

## Methods

### Animals

Twelve week-old male SH rats (Charles River, Raleigh, NC) were housed in plastic cages (one per cage), maintained on a 12-hr light/dark cycle at approximately 22°C and 50% relative humidity in our Association for Assessment and Accreditation of Laboratory Animal Care-approved facility, and held for a minimum of 1 week before telemeter implantation. The Institutional Animal Care and Use Committee of the U.S. Environmental Protection Agency approved all protocols. Food (Prolab RMH 3000; PMI Nutrition International, St. Louis, MO) and water were provided ad libitum, and all rats were grouped by weight. SH rats were selected because previous studies demonstrated exaggerated sensitivity to the effects of air pollution compared to rats with normal blood pressure [20,45,46].

### Telemeter implantation

Animals (SH rats; n = 7 per group) were anesthetized with ketamine/xylazine (80 mg/ml ketamine HCL and 12 mg/ml xylazine HCL; 1 ml/kg i.p.; Sigma Chemical Co., St. Louis, MO), and were implanted with radiotelemeters (Model TA11CTA-F40; Data Science International, Inc., St. Paul, MN) in the abdominal cavity as previously described [47]. Electrode leads were guided through incisions made in the abdominal musculature. Leads were tunneled subcutaneously and secured in a lead II configuration. Body heat was maintained during and after surgery using a heating pad. Animals recovered for two weeks after surgery before inhalation studies.

### Experimental design

SH rats (n = 6/group) surgically implanted with biopotential telemeters were exposed once for 4 hours to concentrated ambient particulate matter (target concentration 150 μg/m^3^) with or without 0.2 ppm ozone. To compare the effects of season on health effects of this multipollutant mixture, exposures were conducted in the summer (August, 2011) and winter (February and March, 2012) seasons. Telemetered signals included body temperature, ECG, heart rate, and activity and were monitored before, during, and after exposure in conscious, unrestrained rats. All telemetered rats were euthanized one day after final exposure. Aconitine challenge was performed to assess sensitivity to the triggering of arrhythmia in a separate cohort of concurrently exposed rats one day after exposure.

### Exposure schedule

A series of particulate matter and O_3_ exposures were conducted in summer of 2011 and winter 2011–12. The study protocol included single 4-hour exposures for CAPs only, CAPs + O_3_, O_3_ only, and filtered air control groups. Upstream of the CAPs only chamber, air passed through a silica gel dryer that reacted with ambient O_3_ and helped keep background O_3_ levels in the CAPs only chamber at levels similar to the filtered air control chamber O_3_ levels. Animals were allowed 15 to 30 minutes to acclimate to the system’s noise and chamber environment then telemetry background measurements were performed for ~45 min. Exposures were initiated at about 7:30 am and continued until 11:30 am for each animal group. For all exposures, initial target PM_2.5_ concentration was ~150 to 200 ug/m^3^ while the O_3_ target concentration was 200 ppb. To facilitate telemetric monitoring within each chamber CAPs and CAPs + O_3_ groups during the summer exposures were split into two cohorts and exposed on either 8/17/11 or 8/18/11. The O_3_ only and air groups were also split into two cohorts and exposed on either 8/23/11 or 8/24/11. Winter CAPs and CAPs + O_3_ exposures were carried out on 2/17/12, 2/18/12, and 3/7/12. Winter O_3_ only and air exposures took place on 2/23/12 and 2/24/12. Each animal group was acclimated to the exposure system and laboratory environmental noises by housing in an exposure chamber with room and CAPs systems operated to produce background noise for a minimum of 1 hour a day for 2 days prior to the exposures.

### Ambient weather patterns

Studies involving exposures of animals to concentrated ambient air particles involve sampling outside air in real-time. Local weather conditions and patterns can have a dramatic effect on these real time studies. Ambient weather conditions were monitored during each exposure using a weather station mounted on the roof (Vantage Pro II, Davis Instruments, Hayward, CA) of the U.S. EPA exposure facility in Durham, NC. Data collected included temperature, relative humidity, dew point, barometric pressure, wind speed and direction, rainfall, and other variables providing information about the air mass being drawn from outside into the system during exposures. Data recorded during the times animals were resident in chambers were averaged to produce individual exposure values. Daily values were then averaged to generate group average ambient weather conditions. Exposure group averages are included in Table 1. Additionally, backward trajectory air mass plots were obtained from on-line resources developed by the National Oceanic and Atmospheric Administration (NOAA). Models queried included Hybrid Single Particle Lagrangian Integrated Trajectory Model (HYSPLIT) using the Real-Time Environmental Applications and Display sYstem (READY) [48], which utilized archived meteorological data to model direction and temporal air mass location. The resultant models indicate the path and location of an air mass in 6 hour increments for the 24 hours local time leading up to each PM exposure.

### Chamber systems

**Figure 9:**
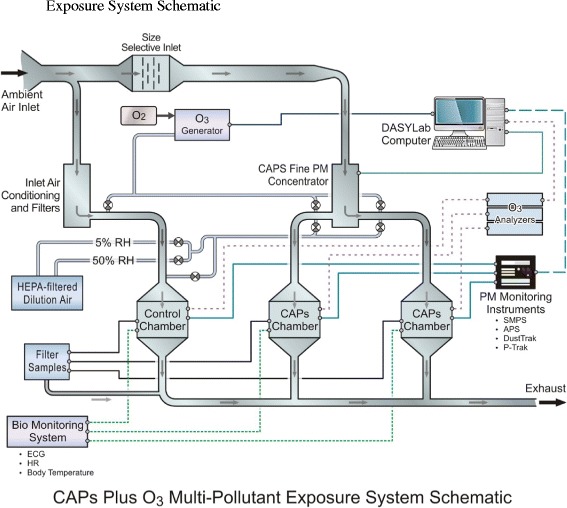
Schematic of concentrated ambient particulates (CAPs) and ozone (O_3_) co-exposure system showing concentrator, O_3_ generator, exposure chambers, and particulate matter (PM) and O_3_ monitoring systems. Receivers were placed within each chamber to monitor electrocardiogram, heart rate and body temperature. Particle concentration and sizing were tracked in real-time using a scanning mobility particle sizer and an aerodynamic particle sizer. Additional aerosol monitors (DustTrak and P-Trak) were used to track PM levels in real-time.

All exposures were conducted in the U. S. EPA’s Research Triangle Park, NC Consolidated Research Facility (CRF), Concentrated Air Particles (CAPs) Laboratory located at 179 T.W. Alexander Dr., RTP, NC. EPA’s CAPs exposure facility accommodated 3 Hinners style, stainless steel and glass, 0.3 m^3^ whole body chambers (Figure 9). Each chamber was modified to expose animals to ozone and clean air as well as CAPs. The wire mesh animal cages were modified and receivers positioned within each chamber to maximize telemeter signal transmission and minimize effects on chamber PM distribution. Two CAPs chambers were connected to the outlet of a PM_2.5_ fine particle concentrator (Harvard Fine Particle Concentrator, Harvard University, Boston, MA; HFPC) [49]. Stainless steel tubing (3” diameter) with quick connecting joints was used to transport Air/PM from the concentrator outlet to the designated chamber(s). Transport tubing was configured to deliver all CAPs to a single chamber, split concentrated PM between two chambers, or to totally bypass chambers allowing for gas pollutant or clean air operation. Chamber temperature, relative humidity, air flow, and static pressure and test agent concentrations for PM and O_3_ were continuously monitored, displayed, and recorded by the lab’s computerized electronic data acquisition system. Clean, charcoal and HEPA filtered, and conditioned dilution air was available from two sources (Core Inhalator System (CIS) supply air, DP = ~55 F, 50% relative humidity (RH), and Medical Grade Air, DP = ~ − 20 F, ~5% RH). The two dilution air streams were blended to regulate chamber RH and peak PM concentrations. Chamber environmental data, PM, and O_3_ concentrations from multiple exposures within each group were averaged to produce mean values for each exposure group.

### PM concentrating system and PM monitoring and control

Outside ambient air containing PM was drawn into the system inlet via a stainless steel duct. The air/PM mixture passed through a custom Size Selective Inlet (SSI) (2 sizing stages: 10 μm and 2.5 μm) (Harvard School of Public Health, Boston, Ma) [50] which removed PM > 2.5 μm. The resulting mixture of Air/PM_2.5_ was transported to the exposure laboratory where it passed through the HFPC operating with 3 stages of slit virtual impactors and outputting ~50 Lpm of Air/CAPs as previously described [28].

CAPs chamber PM concentrations were monitored using both filter weights and real-time electronic instrumentation. Filter samples were collected for all PM exposures to provide gravimetric mass concentrations and for subsequent chemical analysis. Samples included: Teflon 47 mm (Pall Corp., TeFlo, R2PJ047, 2 μm pore) and 37 mm (Pall Corp., TeFlo, R2PJ037, 2 μm pore), Quartz (Millipore, Quartz, AQFA04700, 47 mm), and Nylon (PALL, Nylasorb, 66509, 47 mm) filters. Particle concentration and sizing were tracked in real-time using scanning mobility particle sizer (SMPS, TSI Inc., St. Paul, MN) and aerodynamic particle sizer (APS, model 3321, TSI Inc., St. Paul, MN). PM data were analyzed and combined using particle data analytical software (Data Merge, ver. 1.0.1, TSI Inc, St. Paul, MN) to provide composite concentrations and particle size distributions representative of the average chamber PM. Data from multiple exposures was combined and averaged to provide concentrations and particle size distributions for each group. Additional aerosol monitors (DustTrak (DT), model 8520, TSI Inc., St. Paul, MN and P-Trak, model 8525, TSI Inc., St. Paul, MN) were used to track PM levels in real time. Peak PM concentrations, chamber pressures, and RH were controlled by regulating injection of conditioned, filtered dilution air upstream of the chamber inlets. Filtered and conditioned air supplied to the O_3_ and Air Control groups contained only trace levels of PM with insufficient sample available for weighing or chemical analysis, therefore, no PM samples were collected from those exposures.

### Ozone generation, monitoring, and control

Source O_3_ was generated using O_2_ from compressed gas cylinders fed through a silent arc O_3_ generator (OREC, Osmonics Corp.). All wetted surfaces of the O_3_ generating and monitoring systems were either stainless steel or Teflon. Flow through the generator was modulated so an excess of gas was always flowing to keep stock O_3_ concentrations stable during exposures. Excess O_3_ gas was continuously vented into the lab fume hood exhaust. Chamber O_3_ supply flow was controlled to establish and maintain target O_3_ concentration. Mass flow controllers (MFC) (Tylan Corp., model FC260) were used to control both excess and chamber O_3_ flows. Manual and automated MFC command signals were regulated by the CAPs lab data acquisition system (DAS) running data acquisition and control software (DASYLab Pro, version 9, MCC). O_3_ was injected counter current into the chamber inlet air duct upstream of the dilution air inlet to enhance mixing prior to entering the chamber. O_3_ concentration was manually and/or automatically regulated via a feedback loop. Real-time O_3_ concentrations for each group, including Air Controls, were monitored during all exposures. A continuous O_3_ analyzer (TECO, model 49, Thermo Electron Co.,) measured chamber O_3_ levels in the animal breathing zone and provided concentration signals to the laboratory’s data system. Our laboratory operated under a strict quality assurance plan for all exposures. For the duration of the study, O_3_ monitoring was performed to manufacturer’s and EPA guidelines and under non-condensing conditions. The O_3_ Gas analyzer was calibrated using a certified EPA/National Institute of Standards and Technology O_3_ transfer standard prior to each study segment with routine zero and span checks and post study calibration verifications. The analyzer was preconditioned by continuous sampling of lab air at 72 F / 50% RH. Clean, conditioned dilution air regulated chamber RH and PM levels (therefore, O3 sample RH) which measured in the range 42.6% to 56.5% daily average during all exposures (Table 1). O_3_ sampling system included an inline PM filter (Teflon 47 mm, Pall Corp., TeFlo, R2PJ047, 2 μm pore). The DAS monitored, displayed, recorded, and controlled chamber O_3_ concentration and other system operating variables such as temperature, RH, static pressure, and chamber exhaust flow. Chamber O_3_ concentration was controlled for the duration of each exposure to provide a target final average concentration of ~200 ppb for each run. During exposures, O_3_ flow control was periodically set to manual and maintained at steady state while the O_3_ analyzer was briefly cycled to monitor background O_3_ chambers (CAPs only or Air Controls). After background levels were determined, O_3_ monitoring returned to the O_3_ (or CAPs + O_3)_ exposure chamber under DAS automated control. Outside air passing through the HFPC/CAPs PM concentrator contained naturally occurring ambient O_3_. During CAPs only exposures it was desirable to eliminate this naturally occurring O_3_ to the extent possible without affecting CAPs chamber PM levels. Upstream of the CAPs only chamber, air passed through a silica gel dryer that reacted with ambient O_3_ and helped keep background O_3_ levels in the CAPs only chamber at levels similar to the filtered air control chamber O_3_ levels.

### PM organic and elemental chemical analysis

Organic and elemental carbon contents of CAPs PM collected on quartz filters during exposure were measured using National Institute of Occupational Health (NIOSH) Method 5040 [51]. NIOSH 5040 is a thermo-optical method, based on sequential pyrolytic vaporization and detection of the carbon fractions. Elemental analysis was performed on aqua regia digests of CAP collected on Teflon filters. Prior to digestion, filter media were removed from their polymethylpentene support rings using Teflon blades. Filters were then submerged in 1 mL of concentrated hydrochloric acid (Optima grade, Fisher Scientific) in cleaned 15-ml polypropylene centrifuge tubes (part number 05-539-5, Fisher Scientific; washed in 1% Triton X-100, rinsed with ultrapure deionized water (Milli-Q, Millipore Corporation) and dried in a Class 100 cleanbench). Tubes were sonicated three times in an ultrasonic bath (Model TI-H15, Elma GmbH&Co, Singen Germany) at a frequency of 25 kHz at 200 W for 30 min at 50C°. After each sonication, tubes were cooled to room temperature and vented in a fume hood. After the third sonication and venting, tubes were heated in an oven overnight at 60C°. The next morning, 0.33 mL of concentrated nitric acid (Optima grade, Fisher Scientific) was added to the tubes to form 3:1 aqua regia, and the same sonication and overnight heating procedure was performed as the preceding day. The following morning, 10 ml of ultrapure water was added, and the teflon filters removed from the tubes using cleaned Teflon forceps. Samples were then analyzed by high-resolution inductively coupled plasma-mass spectrometry (HR-ICP-MS, Element 2, Thermo Scientific).

### Source apportionment analysis

Sources contributing to the CAPS and CAPS + O3 mixtures during the summer and winter exposures were quantified using the EPA Chemical Mass Balance Model (CMB 8.2; US EPA, 2004) [19]. The CMB estimates PM sources using a weighted least squares regression. Input data included elements, sulfate, organic carbon and elemental carbon concentrations and uncertainties as well as measured source profile concentrations and uncertainties. The road dust and brake wear profiles were obtained from Hildemann et al. [52] and the mobile source profile (which includes gasoline and diesel emissions) was obtained from Maykut et al. [53]. The wood combustion profile was obtained from Fine et al. [54]. The secondary sulfate and marine salt profiles were obtained from the EPA SPECIATE 4.4 Database. The same profiles and species were used for the summer and winter exposure mixtures.

### Radiotelemetry data acquisition

Radiotelemetry methodology (Data Sciences International, Inc.) allowed constant monitoring of electrocardiographic data in conscious and unrestrained rats from implantation until sacrifice. Electrocardiographic data was monitored by remote receivers (DataART3.01; Data Sciences International, Inc.) positioned under the home cages within the animal facility, and under the exposure cages within the exposure chambers. In home cages, sixty-second segments of ECG waveforms were acquired and saved at 15-minute intervals from surgical recovery through sacrifice not including the exposure period. Pre-exposure baseline data was collected from home cages, as well as a 45 min baseline in exposure cages after acclimation for one hour. During the 4 hr exposure, sixty-second segments were acquired and saved at 5-minute intervals. After exposure, rats were monitored in home cages until the next exposure. All rats were monitored until the beginning of necropsy, approximately 18 hrs after exposure. HR was automatically obtained from the ECG waveforms with data acquisition software (DataART3.01; Data Sciences International, Inc.).

### Electrocardiogram, arrhythmia identification and heart rate variability analysis

ECGAuto software (EMKA Technologies, Falls Church, VA) was used for automated analyses of ECG wave amplitudes and segment durations and areas, as well as for the visual identification and enumeration of arrhythmias and HRV analysis. Several parameters were determined for each ECG waveform: heart rate; PR interval; R amplitude; QRS duration, amplitude, and area; ST interval, amplitude, and area; and T-wave amplitude and area; QT interval, Bazett’s heart rate–corrected QT interval (QTc). ECG and HRV parameters were quantified in the immediate 6-hour period after exposure and compared to time-matched data before exposure while the rats were unrestrained in their home cages. ECG parameters during exposure were analyzed relative to baseline (45 min recordings while in the exposure chambers immediately before the beginning of exposure).

Cardiac arrhythmic events were identified in part by using the Lambeth conventions [55] as a guideline for the identification of arrhythmias in rats. Arrhythmias were identified as atrial premature beats (APB), ventricular premature beats (VPB), sinoatrial block (SAB), atrioventricular block (AVB), or ventricular tachycardia (VT). Arrhythmias were quantified and totaled during the 4 hour exposure period and during the 6 hour period after exposure and compared to pre-exposure counts. Total arrhythmia counts during exposure were quantified (total of 48 one-minute segments during 4 h exposure period).

For the analysis of HRV, rhythms were thoroughly inspected to identify and exclude arrhythmias, artifacts, and 1-min ECG waveforms lacking distinguishable R waves for more than 30 sec. The analysis of HRV generated HR and time-domain measures, including mean time between adjacent QRS complex peaks (the RR interval), a standard deviation of the RR interval (SDNN), SDNN normalized for the effects of heart rate [SDNN/(RR interval x 100)], and the square root of the mean of squared differences of adjacent RR intervals (RMSSD). The SDNN represents overall HRV, whereas RMSSD represents parasympathetic influence over HR. The analysis of HRV also calculated frequency domain parameters including the LF and HF, and the ratio of these two frequency domains (LF/HF). LF is generally believed to represent a combination of sympathetic and parasympathetic tones, whereas HF indicates cardiac vagal (parasympathetic) tone, and LF/HF serves as an index of sympathovagal balance.

### Aconitine challenge

One day after exposure to CAPs, O_3_, CAPs + O_3_, or filtered air, a separate cohort of animals were anesthetized with urethane (1.5 g/kg, *ip*) and underwent the aconitine challenge; supplemental doses of the anesthetic were administered intravenously when necessary to abolish pain reflex. Animal body temperature was maintained at ~36°C with a heating pad. The left jugular vein was cannulated with P.E. 50 polyethylene tubing for the administration of aconitine. Ten μg/ml aconitine was continuously infused at a speed of 0.2 ml/min while ECG was continuously monitored and timed. Sensitivity to arrhythmia was measured as the threshold dose of aconitine required to produce VPBs, VT, and VF, and was calculated using the following formula:

$$ \mathrm{Threshold}\ \mathrm{dose}\ \left(\upmu \mathrm{g}/\mathrm{kg}\right)\ \mathrm{f}\mathrm{o}\mathrm{r}\ \mathrm{arrhythmia} = 10\upmu \mathrm{g}/\mathrm{ml}\ \mathrm{x}\ 0.2\mathrm{ml}/ \min \times \mathrm{time}\ \mathrm{r}\mathrm{equired}\ \mathrm{f}\mathrm{o}\mathrm{r}\ \mathrm{inducing}\ \mathrm{arrhythmia}\ \left( \min \right)/\mathrm{body}\ \mathrm{weight}\ \left(\mathrm{kg}\right) $$

### Necropsy, blood collection and lung lavage

Rats were deeply anesthetized with i.p. injection of Euthasol (200 mg/kg sodium pentobarbital and 25 mg/kg phenytoin; Virbac Animal Health, Ft. Worth Texas) one day after exposure. Blood samples were collected from the abdominal aorta. The trachea was cannulated and the right lung (except for the caudal lobe) was lavaged with a total volume of 20 ml/kg of Ca^2+^, Mg^2+^, and phenol red-free Dulbecco’s phosphate buffered saline (SAFC Biosciences, Lenexa MD) divided into 2 equal aliquots. The caudal lobe was collected for RNA analysis. Cytospins and cells differentials on lavaged cell samples, assays for total protein (Thermo Fisher Diagnostics, Rockford, IL), albumin (Diasorin, Stillwater, MN), LDH (Thermo DMA, Louisville, CO), NAG (Roche Diagnostics, Mannheim, Germany) superoxide dismutase (Randox Laboratories LTD Crumlin, CO), glutathione peroxidase, and glutathione S-transferase (Glathione peroxidase and transferase were based on an in-house automated analysis [56]) on lavage supernatants, serum C-reactive protein and fibrinogen (Diasorin, Stillwater, MN), creatine kinase (Fisher Diagnostics, Middletown, VA), sorbitol dehyrdogenase and creatinine (Sekisui Diagnostics, Charlottetown Prince Edward Island, Canada), high density (HDL) and low density (LDL) lipoprotein cholesterol and plasma angiotensin converting enzyme (Fisher Diagnostics, Middletown, VA) were conducted as previously described [20].

### Statistics

The statistical analyses of ECG, HRV, and biochemical and inflammatory data in this study were performed using SAS software version 9.2 (SAS Institute Inc, Cary, NC). We used PROC MIXED of SAS because it offers greater flexibility for the modeling of repeated measures data than PROC GLM. It is also suitable for analysis of large, unbalanced data with missing data at random. A linear mixed model with restricted maximum-likelihood estimation analysis, least squares means and repeated measures ANOVA (analysis of variance) was used to determine which TIME*TRT interactions were statistically significant between baseline and exposure. Multiple comparison adjustment for the *p* values and confidence limits for the differences between the least squares means was done using adjust = Tukey HSD (Honest Significant Difference) test.

## Additional files

Additional file 1: Table S1. Average source contributions for summer and winter exposures in μg/m^3^. Percent of mass in parenthesis. Additional file 2: Figure S1. Average source contributions for summer and winter Exposures. Additional file 3: Table S2. Source contributions for summer exposures by exposure day in μg/m^3^ (standard deviation in parenthesis). Additional file 4: Table S3. Source contributions for winter exposures by exposure day in μg/m^3^ (standard deviation in parenthesis). Additional file 5: Table S4. Heart Rate Variability data before and after exposure.

## Abbreviations

ANOVA: Analysis of variance
APB: Atrial premature beat
AVB: Atrioventricular block
Cal factor: Calibration factor
CAPs: Concentrated ambient particulates
CIS: Core inhalation system
CMB: Chemical mass balance
CRF: Consolidated research facility
CuZn SOD: Copper zinc superoxide dismutase
DAS: Data acquisition system
DP: Dew point
ECG: Electrocardiogram
GST: Glutathione S-transferase
HDL: High density lipoprotein
HF: High frequency
HFPC: Harvard fine particle concentrator
HR: Heart rate
HRV: Heart rate variability
HSD: Honest significant difference
HYSPLIT: Hybrid single particle lagrangian integrated trajectory
LDH: Lactate dehydrogenase
LDL: Low density lipoprotein
LF: Low frequency
LF/HF: Ratio of low frequency domain to high frequency domain
MFC: Mass flow controller
NAG: N-Acetyl-D-glucosaminidase
NIOSH: National Institute of Occupational Safety and Health
NOAA: National Oceanic and Atmospheric Administration
O_3_: Ozone
PM: Particulate matter
PM_2.5_: Particulate matter with an aerodynamic diameter less than 2.5 microns
READY: Real-time environmental applications and display sYstem
RH: Relative humidity
RMSSD: Root means squared of successive differences
SDNN: Standard deviation of the time between normal-to-normal beats
SH: Spontaneously hypertensive
SSI: Size selective inlet
TRP: Transient receptor potential
US EPA: United States Environmental Protection Agency
UTC: Universal Time Coordinated
VF: Ventricular fibrillation
VPB: Ventricular premature beat
VT: Ventricular tachycardia
