# Supplementary material for: Cardiac effects of seasonal ambient particulate matter and ozone co-exposure in rats
Source: Part Fibre Toxicol. 2015 May 6;12:12. doi: 10.1186/s12989-015-0087-3 (PMC4419498; doi:10.1186/s12989-015-0087-3)
Supplement: Additional file 1: Table S1. — Average source contributions for summer and winter exposures in μg/m3. Percent of mass in parenthesis. [file 12989_2015_87_MOESM1_ESM.pdf]

Farraj et al Additional File 1:

Table A1: Average source contributions for summer and winter exposures in μg/m^3. Percent of mass in parenthesis.

	**Summer Exposures**		**Winter Exposures**	
**Source**	CAPS	CAPS+O_3	CAPS	CAPS+O_3
Mobile Sources	23.89 (14.2)	24.47 (14.4)	14.28 (15.9)	13.36 (17.0)
Brake Wear	0.66 (0.39)	0.20 (0.12)	0.30 (0.34)	0.25 (0.32)
Road Dust	4.58 (2.7)	3.66 (2.1)	5.32 (5.9)	4.67 (6.0)
Wood Combustion	20.47 (12.1)	31.64 (18.6)	26.71 (29.7)	21.27 (27.1)
Marine Salt	1.02 (0.61)	1.43 (0.84)	3.52 (3.9)	3.14 (4.0)
Secondary Sulfate	61.93 (36.7)	68.53 (40.3)	24.22 (26.9)	18.48 (23.6)
Unidentified/Other	56.08 (33.3)	40.27 (23.7)	15.72 (17.5)	17.31 (22.1)
