# Supplementary material for: Cardiac effects of seasonal ambient particulate matter and ozone co-exposure in rats
Source: Part Fibre Toxicol. 2015 May 6;12:12. doi: 10.1186/s12989-015-0087-3 (PMC4419498; doi:10.1186/s12989-015-0087-3)
Supplement: Additional file 2: Figure S1. — Average source contributions for summer and winter Exposures. [file 12989_2015_87_MOESM2_ESM.pdf]

Farraj et al Additional File 2

Figure A1: Average source contributions for summer and winter exposures

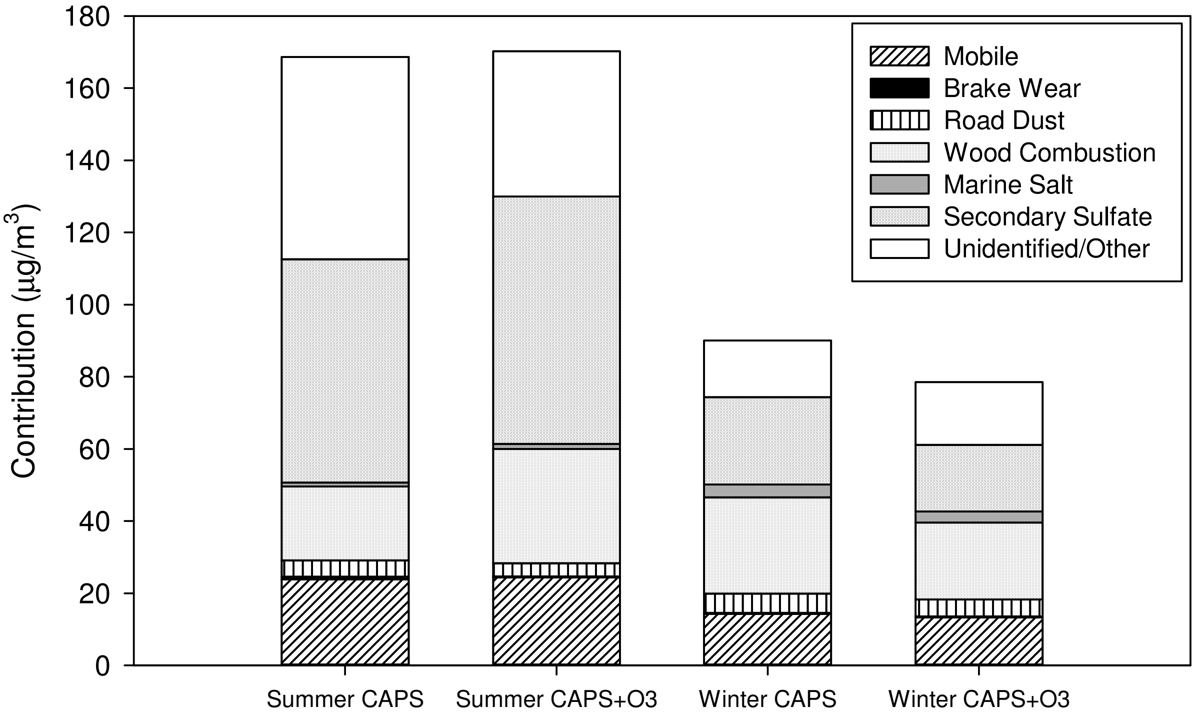

Figure A1: Stacked vertical bar representation of sources contributing to the CAPS and CAPS+O_3 mixtures during the summer and winter exposures.
