# Supplementary material for: Cardiac effects of seasonal ambient particulate matter and ozone co-exposure in rats
Source: Part Fibre Toxicol. 2015 May 6;12:12. doi: 10.1186/s12989-015-0087-3 (PMC4419498; doi:10.1186/s12989-015-0087-3)
Supplement: Additional file 3: Table S2. — Source contributions for summer exposures by exposure day in μg/m3 (standard deviation in parenthesis). [file 12989_2015_87_MOESM3_ESM.pdf]

Farraj et al Additional File 3:

Table A2: Source contributions for summer exposures by exposure day in μg/m^3 (standard deviation in parenthesis).

**Source**	**Summer Exposures**		
	CAPS 8/17/2011	CAPS 8/18/2011	CAPS+O_3 8/18/2011
Mobile Sources	35.51 (6.95)	12.27 (2.46)	24.47 (4.81)
Brake Wear	1.15 (0.17)	0.16 (0.03)	0.20 (0.03)
Road Dust	5.37 (1.93)	3.79 (0.73)	3.66 (1.33)
Wood Combustion	13.74 (3.86)	27.21 (3.01)	31.64 (4.03)
Marine Salt	0.88 (0.10)	1.16 (0.14)	1.43 (0.18)
Secondary Sulfate	67.23 (9.81)	56.63 (8.11)	68.53 (9.89)
