# Supplementary material for: Cardiac effects of seasonal ambient particulate matter and ozone co-exposure in rats
Source: Part Fibre Toxicol. 2015 May 6;12:12. doi: 10.1186/s12989-015-0087-3 (PMC4419498; doi:10.1186/s12989-015-0087-3)
Supplement: Additional file 4: Table S3. — Source contributions for winter exposures by exposure day in μg/m3 (standard deviation in parenthesis). [file 12989_2015_87_MOESM4_ESM.pdf]

Farraj et al Additional File 4:

Table A3: Source contributions for winter exposures by exposure day in μg/m^3 (standard deviation in parenthesis).

**Source**	**Winter Exposures**					
	CAPS 2/28/2012	CAPS 2/29/2012	CAPS 3/7/2012	CAPS+O_3 2/28/2012	CAPS+O_3 2/29/2012	CAPS+O_3 3/7/2012
Mobile Sources	19.54 (3.86)	8.64 (1.75)	14.65 (3.07)	18.52 (3.64)	7.42 (1.51)	14.14 (2.93)
Brake Wear	0.36 (0.06)	0.15 (0.02)	0.40 (0.07)	0.33 (0.05)	0.13 (0.02)	0.29 (0.05)
Road Dust	4.42 (1.09)	1.80 (0.48)	9.74 (1.18)	3.26 (1.01)	1.77 (0.42)	8.98 (1.08)
Wood Combustion	19.13 (2.88)	27.31 (2.88)	33.68 (4.36)	16.90 (2.59)	21.75 (2.36)	25.16 (3.47)
Marine Salt	1.23 (0.14)	4.45 (0.53)	4.87 (0.57)	1.05 (0.12)	4.08 (0.48)	4.28 (0.51)
Secondary Sulfate	20.38 (3.09)	23.21 (3.40)	29.08 (4.30)	14.03 (2.20)	16.71 (2.48)	24.71 (3.67)
