# Supplementary material for: Cardiac effects of seasonal ambient particulate matter and ozone co-exposure in rats
Source: Part Fibre Toxicol. 2015 May 6;12:12. doi: 10.1186/s12989-015-0087-3 (PMC4419498; doi:10.1186/s12989-015-0087-3)
Supplement: Additional file 5: Table S4. — Heart Rate Variability data before and after exposure. [file 12989_2015_87_MOESM5_ESM.pdf]

Farraj et al Additional File 5:

Table A4: Heart Rate Variability data before and after exposure

	Summer Exposures								Winter Exposures							
	Air		CAPs		O_3		CAPs+O_3		Air		CAPs		O_3		CAPs+O_3	
	Pre	Post	Pre	Post	Pre	Post	Pre	Post	Pre	Post	Pre	Post	Pre	Post	Pre	Post
**RMSSD (msec)**	3.5±0	3.6±0	5.5±0	5.1±0	3.3±0	3.7±0	4.5±0	3.6±0[a]	3.4±0	3.5±0	3.4±0	3.4±0	3.9±0	3.6±0	4.1±0	4.2±0
**LF/HF(msec)**	1.1±0	1.0±0	0.9±0	1.0±0	0.7±0	1.2±0[b]	0.7±0	0.9±0[b]	1.3±0	0.9±0	0.8±0	1.0±0	0.9±0	1.4±0	0.9±0	1.4±0

Note: Data represent means during the immediate 6-hour period following exposure (Post) and the corresponding time-matched pre-exposure period (Pre).
a – Significant decrease from pre-exposure value; b – significant increase from pre-exposure value.
